# Characterization of faecal and caecal microbiota of free-ranging black-tailed prairie dogs (*Cynomys ludovicianus*) using high-throughput sequencing of the V4 region of the 16S rRNA gene

**DOI:** 10.1093/conphys/coab042

**Published:** 2021-06-15

**Authors:** Tess A Rooney, David Eshar, Charles Lee, J Scott Weese

**Affiliations:** 1 Department of Clinical Sciences, College of Veterinary Medicine, Kansas State University, 1800 Denison Avenue, Manhattan, KS 66506, USA; 2 Animal Sciences and Industry, College of Agriculture, Kansas State University, 1530 Mid-Campus Drive North, Manhattan, KS 66506, USA; 3 Ontario Veterinary College, University of Guelph, 50 Stone Road E., Guelph, Ontario N1G 2W1, Canada

**Keywords:** 16 s rRNA, black-tailed prairie dog, Cynomys ludovicianus, faecal and caecal, microbiome, next-generation sequencing

## Abstract

Black-tailed prairie dogs (*Cynomys ludovicianus*) are keystone species within their grassland ecosystems; their population stability affects a multitude of other species. The goals of this study were to explore, describe and compare the bacterial communities in caecal and hard faecal samples from free-ranging black-tailed prairie dogs (*n* = 36) from KS, USA, using high-throughput sequencing of the V4 region of the 16S rRNA gene and to compare sex and geographic locations. A total of 22 paired faecal and caecal samples were collected post-mortem from free-ranging black-tailed prairie dogs from 5 different geographical locations. The results revealed that the microbiota of both faecal and caecal samples were dominated by the phylum Firmicutes (genera belonging to the Clostridiales order). There was significantly greater richness in faecal compared with caecal samples. There were significant differences between the 5 different geographic regions (*P* < 0.001), specifically in the relative abundances of genera. There were differences in rare members of the microbiome between faecal samples from male and female prairie dogs but with no significant impact on overall community structure. This study provides novel data and expands our knowledge about the gastrointestinal microbiome composition of free-ranging black-tailed prairie dogs, which has potential to inform conservation efforts and improve their captive management.

## Teaser

This study characterized the faecal and caecal microbiota of free-ranging black-tailed prairie dogs in KS, USA, with consideration to their sex and geographic location.

## Introduction

The prairie dog (*Cynomys ludovicianus*) is a medium-sized, colonial, burrowing rodent in the order Rodentia, suborder Sciuromorpha and family Sciuridae (Hoogland, 2006). Their habitat, within the grasslands of western North America, spans the full length of the country from Canada to northern Mexico and includes predominantly mixed-prairie and shortgrass steppe, as well as desert grasslands and shrublands (Hoogland, 2006). Prairie dogs are recognized as keystone species in this ecosystem due to their effect on topsoil composition (via behaviour and defecation habits), creation of burrows that other species use and the fact that they serve as important prey species for endangered predators, including the black-footed ferret (*Mustela nigripes*) (Hoogland, 2006). In their natural habitat, prairie dogs are generally herbivorous, consuming grasses, herbs and weeds, with a couple of notable exceptions: wild prairie dogs will consume faeces from American bison (*Bison bison*), a couple of specific insect species and may cannibalize deceased or neonatal prairie dogs (Miller and Fowler, 2014).

The intestinal microbiota has gained attention in both human and veterinary medicine due to its crucial impacts on the overall health of its host organism. The commensal organisms of the gastrointestinal tract aid in the development of innate and adaptive immunity, produce vitamins and protect against pathogens (Dieterich *et al.*, 2018). They help to digest fibre, producing short-chain fatty acids as a byproduct of the fermentative metabolic process (Honneffer *et al.*, 2014). Besides supplying metabolic energy demands, these short-chain fatty acids have proposed impacts on physiology and immune system function (Honneffer *et al.*, 2014; Koh *et al.*, 2016). The bacterial populations that promote these important functions are responsive to diet, which has been shown to rapidly and consistently alter the community structure and relative proportions of the gut microbiota (Lawrence *et al.*, 2014). One can conclude that it is important to maintain a healthy gut microbiome in order to maintain overall health and to prevent certain types of gastrointestinal pathology (Eshar *et al.*, 2019). For example, disruption of the microbial community, or dysbiosis, is a common cause of morbidity and mortality in rodent species (Kylie *et al.*, 2018). Studies have been conducted on a variety of species in an attempt to characterize the microbiome and determine the richness of species diversity. Faecal analysis has been used to evaluate the composition of the gut microbiome and the effect of the diet on the microbiome; this method has been previously described in both domestic and non-domestic felids (Zierer *et al.,* 2018, Eshar *et al.*, 2019). However, there may be substantial differences in the functional activity and environment of different regions of the gastrointestinal tract in hindgut fermenters that may affect the microbiome along its course (Tanca *et al.*, 2017). Conservation physiology is a concept that relates organisms’ physiology to broad-scale conservation implications, including a species’ ability to thrive in a given environment (Cooke *et al.,* 2013). A better understanding of the prairie dog microbiome in its natural habitat may inform conservation efforts and improve their captive management.

In recent years, technologies including next-generation sequencing have largely replaced traditional culture-based methods for microbe identification. Thus, microorganisms may be studied as a community, rather than as single organisms in isolation. These technologies allow for a comprehensive characterization of microbial communities and the data obtained provides opportunities to challenge current viewpoints of what constitutes ‘normal’ microbial populations. The faecal microbiome of black-tailed prairie dogs has been previously described based on 10 faecal samples collected from around a single prairie dog burrow from the Janos Biosphere Reserve, Chihuahua, Mexico; the number of individuals contributing to the samples and the sex of the animals were unknown (Pacheco-Torres *et al.*, 2019). The current study evaluated samples from 36 individual prairie dogs, compared sex and geographically distinct groups and compared faecal to caecal samples. The goals of the current study were to explore, describe and compare the bacterial communities in caecal and hard faeces samples from free-ranging black-tailed prairie dogs, using high-throughput sequencing of the V4 region of the 16S rRNA gene and to compare sex and geographic locations.

## Materials and methods

### Study population and sample collection

Faecal and caecal samples were collected from free-ranging black-tailed prairie dogs during the summer (August–September) from five different geographical locations (southern Logan County, western Logan county, Barber County, Stafford County and Stockton County, all in KS, USA). From 14 animals, faecal samples alone were obtained; from 22 animals, both faecal and caecal samples were obtained. The samples were opportunistically collected from legally, freshly killed (gun-shot) prairie dogs, as part of a population control program. The samples were collected immediately after hunting and initially stored in −20°C and later in −70°C until analysis. This study was approved by the Kansas State, College of Veterinary Medicine Ethics Committee (Institutional Animal Care and Use Committee #3448).

### DNA extraction and quality control

All samples were processed at the same time at the Ontario Veterinary College. DNA was extracted from faecal and caecal samples using a commercial kit (E.Z.N.A.) as per manufacturer’s instruction. DNA quantity and quality were assessed via spectrophotometry (NanoDrop). The V4 region of the 16S rRNA gene was amplified, then a second PCR reaction was performed to attach (Caporaso *et al.,* 2010) Illumina universal index sequencing adaptors. After purification with AMPure X (Beckman Coulter Inc.) and assessment of DNA spectrophotometry, DNA quantity was normalized to a final concentration of 2 nM. Sequencing was performed using an Illumina MiSeq (Illumina MiSeq) (2 × 250 chemistry).

### Microbiota assessment and analysis

Sequences were processed and analysed using Mothur (v1.37) (Schloss *et al.*, 2009). Paired-end reads were aligned, then sequences were passed through a series of quality control steps to remove sequences that contained any ambiguous base calls, were not consistent with the target amplicon size (240 bp), contained holopolymers of >8 bp in length or did not align with the correct 16S rRNA gene region. UCHIME was used to identify chimeras (Edgar *et al.*, 2011), which were then removed. Taxonomy was assigned using the RDP classifier (Wang *et al.* 2007). Relative abundances of taxa were compared between groups using Wilcoxon test with adjustment of *P*-values for false discovery rate using the Benjamini–Hochberg technique. Data analyses were performed using JMP 12 SAS and R Core Team (JMP 12 SAS; R Core Team, 2013). Comparison of genera was limited to genera that were among the 40 most abundant genera in the corresponding group, to avoid excessive loss of power following correction for false discovery rate. Comparison of genera between different wild prairie dog groups was performed using Steel–Dwass test. A *P*-value of ≤0.05 was considered significant for all analyses.

Sequences were binned into operational taxonomic units (OTUs) in a de novo (non-database dependent) approach using the average neighbour algorithm. Subsampling was performed to normalize sequence number for subsequent analyses (Gihring *et al.*, 2012). Alpha diversity indices (observed richness, Chao1 estimated richness, inverse Simpson’s diversity, Shannon’s evenness tests) were calculated and compared using Wilcoxon test. Beta-diversity was evaluated through comparison of community membership (classical Jaccard index, a measure of shared OTUs) and structure (Yue and Clayton index of dissimilarity, which considers shared OTUs and their relative abundances), as well as using ANOSIM. Unifrac and analysis of molecular variance were used to compare groups. Community membership and structure were also visualized using principal coordinate analysis. Linear discriminant analysis effect size (LEfSe) (Segata *et al.*, 2011) was used to identify differentially abundant taxa. Samples were also evaluated using the Dirichlet multinomial mixtures method for probabilistic modelling (Holmes *et al.*, 2012). This was used to determine how many different metacommunities (enterotypes) selected sample populations (i.e. faecal versus caecal, male versus female) could be assigned to. This was based on the *K*-value (metacommunity number) that derived the minimum Laplace approximation. The observed learning technique, random forests, was used to determine if a learning-based approach could be used to determine predictive features to accurately identify samples from faecal versus caecal and male versus female samples (Knights *et al.*, 2011). The core microbiota was assessed through identification of OTUs that were present in all faecal or caecal samples at a relative abundance of at least 1%.

## Results

A total of 58 samples from 36 prairie dogs were processed in this study, including males (*n* = 19) and females (*n* = 17). Paired caecal and faecal samples were collected from 22 prairie dogs (*n* = 44 samples) and faecal samples only from 14 prairie dogs. A total of 4 142 277 sequences were retained after processing and quality control filtering, with a median of 67 751 sequences per sample (range, 26 827–112 989; mean, 66 811). A random subsample of 29 323 sequences per sample was selected to normalize sample numbers for analysis for comparison of faecal versus caecal samples and 38 822 sequences were used to compare male versus female free-ranging prairie dogs.

## Faecal **versus** caecal

Paired faecal and caecal samples were available for 22 prairie dogs (*n* = 44 samples). There was a significant difference in observed richness (*P* = 0.026), with greater richness in faecal (median, 2397 OTUs) compared to caecal samples (median, 2237 OTUs). The corresponding difference in estimated richness (Chao1, 3966 vs 3710 OTUs) was not statistically significant but approached significance (*P* = 0.056). There were no differences in diversity (*P* = 0.33) or evenness (*P* = 0.55).

**Figure 1:**
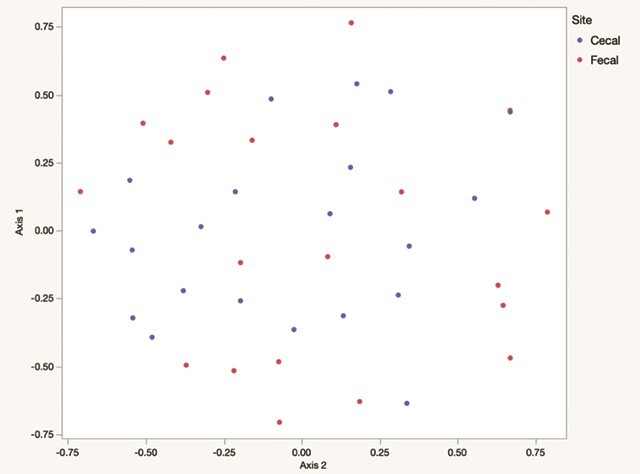
Principle coordinate analysis depicting the community membership of the fecal (green) and caecal (red) microbiomes in 22 free-ranging black-tailed prairie dogs (*C. ludovicianus*), based on the Yue and Clayton index of dissimilarity (samples from the same animal are connected by lines)

There were no differences in community membership (*P* = 0.96) or community structure (*P* = 0.99). Visually, samples clustered within animals. When community membership (Jaccard index) was assessed, 17/21 (81%) of paired samples were closer to each other than any other sample. When relative abundance was included (Yue and Clayton index), 11/21 (52%) caecal–faecal pairs clustered closest to each other. The close relationship between faecal–caecal pairs was also evident by NMDS (Fig. 1). ANOSIM was performed on the faecal/caecal comparison and revealed *P*-values of <0.0001 for the Jaccard index and 0.026 for the Yue and Clayton index of dissimilarity. The ANOSIM *R*-values were 0.085 for Jaccard index comparison and 0.057 for Yue and Clayton index of dissimilarity. Despite the lack of significant differences in studied alpha and beta diversity parameters, random forests successfully classified 15/21 (71%) caecal samples and 16/21 (76%) faecal samples, for an overall error rate of 26%.

**Figure 2:**
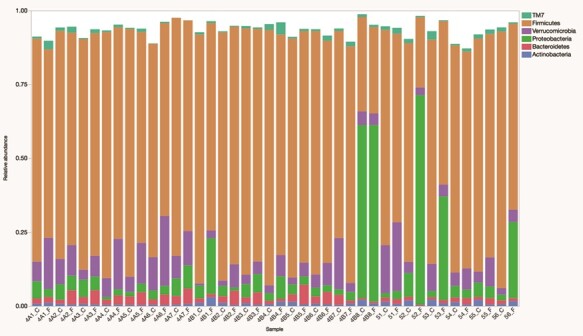
Relative abundances of phyla accounting for a minimum of 1% of sequences in the fecal (_F) and caecal (_C) microbiomes of 22 free-ranging black-tailed prairie dogs (*C. ludovicianus*)

Firmicutes was the most abundant phylum present in both faecal and caecal samples. This phylum was present in the greatest relative abundance in all but three samples, where Proteobacteria dominated. Interestingly, two of those were paired faecal and caecal samples from the same animal (Fig. 2). Overall, 28 different phyla were identified, but only 4 had median relative abundances greater than 1%: Firmicutes (75%), Verrucomicrobia (6.5%), Proteobacteria (4.3%) and Bacteroidetes (1.8%). There were no significant differences in the relative abundances of difference phyla; however, there were numerous differences at lower taxonomic levels. Significant differences among the 40 most abundant members of class through genus are presented in Table 1. Genera with the highest relative abundances are presented in Table 2. The genera that were significantly more common in faecal samples were also identified as enriched via LEfSe (Table 3).

**Table 1:** Median relative abundance (range) of taxa of bacteria that were significantly different in faecal and caecal samples of 22 free-ranging black-tailed prairie dogs (*C. ludovicianus*), in descending order from class to genus

Level	Taxon	Faecal	Caecal	Adjusted *P*-value
Class	Alphaproteobacteria	**0.0056 (0.000629–0.025853)**	0.0014 (0.000375–0.008563)	0.013
	Elusimicrobia	**0.00095** **(0–0.00044)**	0.00015 (0–0.000111)	0.014
Order	Clostridiales	0.57 (0.14835–0.64806)	**0.62 (0.179643–0.720777)**	0.041
	Bacteroidales	**0.017** **(0.002421–0.04339)**	0.0080 (0.004421–0.021204)	0.041
	Unclassified Proteobacteria^a^	0.0086 (0.00221–0.016103)	**0.012** **(0.002964–0.049915)**	0.030
	Unclassified Clostridia^a^	0.010 (0.003879–0.013474)	**0.014** **(0.003198–0.024306)**	0.007
	Unclassified Alphaproteobacteria^a^	**0.0049** **(0.000199–0.010541)**	0.0003 (0.000058–0.001303)	0.0009
	Erysipelotrichales	**0.0020** **(0.000388–0.006536)**	0.0012 (0.000383–0.003292)	0.041
	Unclassified Deltaproteobacteria^a^	**0.00064** **(0.000122–0.001372)**	0.00014 (0.000022–0.000667)	0.0018
Family	Unclassified Alphaproteobacteria	**0.0027** **(0.000199–0.010541)**	0.00029 (0.000058–0.001303)	0.0011
	Coriobacteriaceae	0.0043 (0.001587–0.008125)	**0.0062** **(0.003293–0.019267)**	0.020
	Unclassified Clostridia	0.010 (0.003879- 0.013474)	**0.014 (0.003198–0.024306)**	0.026
Genus	Unclassified Clostridiales^b^	0.010 (0.003879–0.013474)	**0.014 (0.003198–0.024306)**	0.021
	Unclassified Ruminococcaceae^b^	0.098 (0.027951–0.152148)	**0.127 (0.033371–0.17979)**	0.021
	Unclassified Proteobacteria^b^	0.0086	**0.012**	0.044

The significantly higher value is indicated in bold.

a Unclassified order belonging to the higher stated taxonomic level.

b Unclassified genus belonging to the higher stated taxonomic level.

**Table 2:** Median relative abundance of predominant bacterial genera in the faecal and caecal microbiota of 22 free-ranging black-tailed prairie dogs (*C. ludovicianus*)

Cecum		Faeces	
Genus	Median relative abundance	Genus	Median relative abundance
Unclassified Clostridiales^a^	0.23	Unclassified Clostridiales^a^	0.19
Unclassified Ruminococcaceae^a^	0.13	Unclassified Ruminococcaceae^a^	0.098
Unclassified Lachnospiraceae^a^	0.11	Unclassified Firmicutes^a^	0.087
Unclassified Firmicutes^a^	0.094	Unclassified Lachnospiraceae^a^	0.076
*Ruminococcus*	0.026	*Akkermansia*	0.044
*Akkermansia*	0.024	*Ruminococcus*	0.030
*Sporobacter*	0.021	Unclassified Verrucomicrobiaceae^a^	0.020
*Clostridium* cluster IV	0.017	*Sporobacter*	0.014
Unclassified Clostridia^a^	0.014	*Clostridium* cluster IV	0.011
Unclassified Verrucomicrobiaceae^a^	0.012	Unclassified Clostridia^a^	0.010

a Unclassified genus belonging to the higher stated taxonomic level.

**Table 3:** OTUs that were differentially abundant in the faecal versus caecal microbiomes of free-ranging black-tailed prairie dogs (*C. ludovicianus*) with a minimum linear discriminant analysis score of 3

Caecal	Faecal
Unclassified Clostridiales (6 OTUs)^a^	*Escherichia_Shigella*
Unclassified Ruminococcaceae^a^	*Acidaminobacter*
*Lysinibacillus*	*Anaerostipes*
Unclassified Firmicutes^a^	Unclassified Clostridiales^a^
Unclassified Proteobacteria^a^	Unclassified Planococcaceae^a^
*Clostridium* cluster IV (Ruminococcaceae)	Unclassified Lachnospiraceae^a^
Unclassified Clostridia^a^	

a Unclassified genus belonging to the stated higher taxonomic level.

No OTUs were present in all caecal or faecal samples at a relative abundance of at least 1%. *Ruminococcus* was present at that relative abundance threshold in 17/21 (81%) of faecal samples, with an unclassified Ruminococcaceae and unclassified Verrucomicrobiaceae present at that level in 13 (62%) samples. The same three OTUs were identified as predominant members of caecal samples, with the unclassified Ruminococcaceae being present in 17 (81%) caecal samples at that relative abundance and *Ruminococcus* in 15 (71%) and an unclassified Verrucomicrobiaceae in 12 (57%).

## **Comparison of the** faecal **Microbiota of Prairie Dogs from Different Geographic Locations**

**Figure 3:**
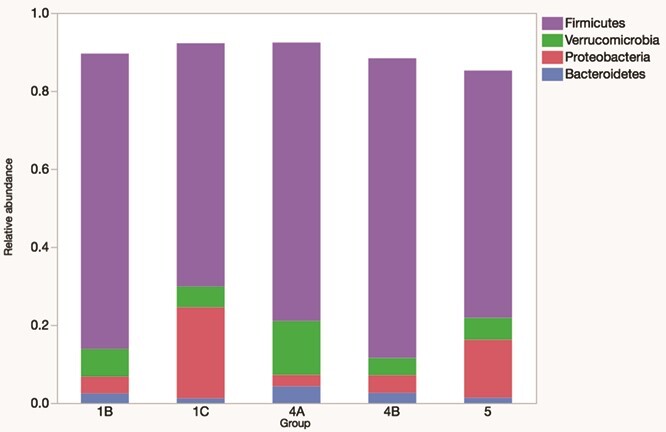
Relative abundances of the main phyla in the faecal microbiome of 22 free-ranging black-tailed prairie dogs (*C. ludovicianus*) from 5 geographic locations

**Figure 4:**
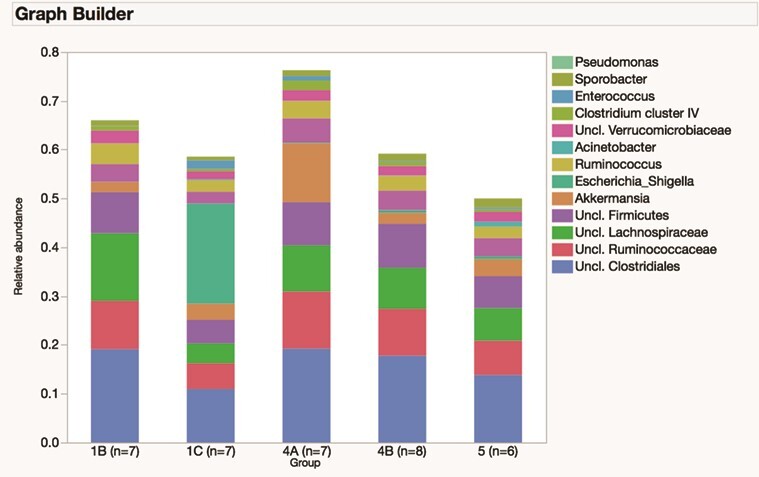
Median relative abundances of predominant genera in the faecal microbiome of 22 free-ranging black-tailed prairie dogs (*C. ludovicianus*) from 5 geographic locations (denoted as 1B, 1C, 4A, 4B and 5)

A total of 36 faecal samples were obtained from prairie dogs belonging to 5 different geographical locations. The number of samples per group is as follows: Southern Logan County, 7; Barber County, 7; Western Logan County, 7; Stockton County, 8; and Stafford County, 7. The community membership and structure faecal microbiota differed significantly between groups (both *P* < 0.001). There were multiple differences in the relative abundances of phyla and genera (Figs 3 and 4).

## **Comparison of the** faecal **Microbiota of Male Versus Female Prairie Dogs**

A total of 36 faecal samples were obtained from prairie dogs of both male (*n* = 19) and female (*n* = 17) sexes. There were no differences in estimated richness (*P* = 0.44), observed richness (*P* = 0.19) and diversity (*P* = 0.23). There were also no differences in the relative abundances of any phyla, classes, orders, families or genera after correction for false discovery rate. There was a significant difference in membership between males and females (*P* = 0.008); yet, there was no difference in community structure (*P* = 0.403), suggesting that there were differences in rare members that did not influence overall community structure. Random forests were poorly able to assign samples to their respective groups, with an error rate of 43%, and samples were assigned to a single metacommunity (enterotype).

## Discussion

This study contributes novel information pertaining to the composition of gastrointestinal microbiota of free-ranging black-tailed prairie dogs. The main findings in this study were the following: (i) the predominant phylum in both faecal and caecal samples was Firmicutes and the most prevalent organisms within this phylum were unclassified Clostridiales organisms; (ii) there were differences in rare members of the microbiota between faecal samples from male and female prairie dogs that had no significant impact on overall community structure; (iii) faecal samples have a greater richness than caecal samples; and (iv) there are significant differences in the abundance of genera across the five geographic groups.

In this study, the predominant phylum identified in the prairie dog microbiota in both faecal and caecal samples was Firmicutes. Within Firmicutes, the most prevalent groupings in both faecal and caecal samples in this study were unclassified organisms in the order Clostridiales. These findings are consistent with the previous study on the faecal microbiota of black-tailed prairie dogs in Mexico (Pacheco-Torres *et al.*, 2019). Firmicutes are described as the most abundant phylum in the gastrointestinal tract of most healthy adult mammals (Kylie *et al.*, 2018), including studies specific to domestic rabbits (*Oryctolagus cuniculi*) and guinea pigs (*Cavia porcellus*), which are both also hind-gut fermenters (Crowley *et al.*, 2017; Kylie *et al.*, 2018; Zhu *et al.*, 2015). Firmicutes is proposed as a critical component of gastrointestinal health, both due to its prevalence across mammalian species and the fact that its relative abundance decreases compared with other phyla during metabolic disorders in numerous species (Kylie *et al.*, 2018). One major difference between our findings and those by Pacheco *et al.* (2019) is that in their study, Bacteroidetes accounted for 9.94% of the microbiota (second-most abundant phyla) and only 1.8% in the present study (fourth-most abundant phyla). Other studies have demonstrated negative associations between obesity and high-calorie diets and Bacteroidetes abundance (Turnbaugh *et al.*, 2006). It is possible that differences in the consumed diets or the testing methodologies between the two prairie dog studies account for the different Bacteroidetes abundance. Otherwise, the findings in the present study are consistent with what has been previously described.

Faecal samples in this study demonstrated a greater difference in richness when compared with caecal samples. Many studies use faecal samples as a representation of the gastrointestinal microbiome, with an ongoing debate as to how well the faecal microbiome represents the microbiome in more proximal sections of the gastrointestinal tract (Costa *et al.*, 2015, Wydeven and Dahlgren, 1982). This is an inherent limitation to many studies due to the invasive nature of obtaining caecal samples. A previous study comparing the stomach and faecal contents of black-tailed prairie dogs remarked that while faecal samples provided a fair reflection of the ingesta in the stomach, the mean number of plant species found per faecal sample was higher than the number found per stomach sample (Wydeven and Dahlgren, 1982). A study pertaining to the gastrointestinal microbiome in domestic horses (*Equus caballus*) demonstrated significant variability in microbial composition between compartments of the gastrointestinal tract (Costa *et al.*, 2015). Despite these differences, it was proposed that in the horse faecal samples are a reasonable representation of the microbial composition of the distal gastrointestinal tract (Costa *et al.*, 2015). In rabbits, conflicting data have been reported as to whether hard faeces are less representative of the caecal microbiome than soft faeces (Michelland *et al.*, 2010a; Michelland *et al.*, 2010b). One study comparing the caecal and faecal bacterial communities of rabbits found that there was little difference between caecal and faecal microbial populations, but that both populations were affected by the stress of a surgical procedure (Michelland *et al.*, 2010a). While there was a significant difference in faecal and caecal samples when ANOSIM was employed, the low *R*-value indicates that the effect was minor, which may explain why there was no significant difference noted when the Jaccard index and Yue and Clayton were used alone. Based on the finding in the present study, faecal samples appear to provide good representation of the caecal microbiome in free-ranging black-tailed prairie dogs.

The faecal samples collected in this study from five different areas in KS, USA, showed significant differences between geographic groups (*P* < 0.001), specifically in the relative abundances of genera. These counties represent distinct ecoregions; high plains, red hills, Arkansas river lowlands and smoky hills that are characterized by different topography, and therefore, support different vegetation (Kansas, 2019). The varied fibre content of the natural vegetation is one possible explanation for the regional differences between the faecal microbiota between groups of free-ranging prairie dogs. Previous studies have suggested that relative proportion and source of dietary fibre affect the microbial makeup of rabbit faeces (Crowley *et al.*, 2017; Kylie *et al.*, 2018). Another study found that between two squirrel species in the same habitat, the squirrel species with a higher fibre-content diet was found to have proportionally more gut microbes that are known to play a role in fibre degradation (Reed *et al.*, 2019). It was beyond the scope of this study to evaluate the plant composition from the different geographic regions and their association with the prairie dog faecal microbiota, but differences in the plants’ nutritional profiles across regions may explain the differences in the prairie dog faecal microbiota. Additionally, the samples in this study were collected during the months of August–September, so future studies may collect samples year-round in order to evaluate potential seasonal variations in the microflora that may coincide with different vegetation that may be available to the prairie dogs.

There are several limitations to the study that should be considered. This study employed 16S rRNA gene sequencing for characterization of the faecal and caecal microbiota. A study by Hatton *et al*. (2017) explored diet’s influence on the arctic ground squirrels’ microbiome and employed both 16S rRNA gene sequencing and metatranscriptomic analysis. That study reported that while there was no significant difference in the relative abundance of the microbes across diet groups based on the 16S rRNA gene sequencing, they discovered using metatranscriptomic analysis that gene transcripts specific to altered growth rate based on environmental changes were different based on dietary grouping (Hatton *et al.*, 2017). These authors commented that changes to the metatranscriptome can be identified earlier than changes to the microbial community itself and may be useful as a tool for predicting impending change to the community composition (Hatton *et al.*, 2017). Metatranscriptomic analysis was also cited as an ideal next step in a study characterizing the caecal microbiome of flying squirrels and would be interesting to employ in future studies on the prairie dog microbiome (Lu *et al.*, 2018).

Additionally, this study only investigated the bacterial microbiota of black-tailed prairie dogs, while it has been well-established that other microorganisms also play crucial roles in gastrointestinal health (Kylie *et al.*, 2018). Fungi and protozoa have been proposed as important players in fibre digestion (Reed *et al.*, 2019) and Kylie *et al.* (2018) proposed that since the virome of other rodents (namely, rabbits) has been implicated in the pathogenesis of gastrointestinal dysbiosis, it should be further explored in future studies. One limitation of next-generation sequencing is that it does not account for whether or not the organisms that it identifies are active, so follow-up into the metabolic pathways of these organisms may represent a reasonable next step in this area of research (Kylie *et al.*, 2018). RNA sequencing is a metatranscriptomic approach to studying microbial gene expression that allows detection of all of the RNA expressed by a microbe and characterization in terms of their role in metabolic pathways, and may be a useful tool in future studies (Shakya *et al.*, 2019).

Finally, this study only evaluated the bacterial microbiota of free-ranging black-tailed prairie dogs, which may differ from captive prairie dogs that are housed under specific management regimes and with provision of diets. Characterizing the microbiome of captive prairie dogs and comparing the results with those found in this study would be helpful for determining what, if any, differences are present between free-ranging and captive populations and how management strategies and different diet compositions may influence the microbiome.

## Conclusions

This study characterized the composition of faecal and caecal bacterial microbiomes of free-ranging black-tailed prairie dog in KS, USA. The results demonstrated that geography may affect the microbiome, that faecal samples have a relatively similar composition compared with caecal samples and that there were differences in rare members between male and female prairie dogs that had no significant impact on overall community structure. Further studies employing metatranscriptomic approaches are indicated to better characterize the biologically active microbes within the community.

## Funding

There is no funding to report.
